# Threshold temperatures and thermal requirements of black soldier fly *Hermetia illucens*: Implications for mass production

**DOI:** 10.1371/journal.pone.0206097

**Published:** 2018-11-01

**Authors:** Shaphan Yong Chia, Chrysantus Mbi Tanga, Fathiya M. Khamis, Samira A. Mohamed, Daisy Salifu, Subramanian Sevgan, Komi K. M. Fiaboe, Saliou Niassy, Joop J. A. van Loon, Marcel Dicke, Sunday Ekesi

**Affiliations:** 1 Laboratory of Entomology, Plant Sciences Group, Wageningen University, Wageningen, The Netherlands; 2 International Centre of Insect Physiology and Ecology (*icipe*), Nairobi, Kenya; Universita degli Studi della Basilicata, ITALY

## Abstract

Efforts to recycle organic wastes using black soldier fly (BSF) *Hermetia illucens* into high-nutrient biomass that constitutes a sustainable fat (biodiesel) and high-quality protein ingredient in animal feeds have recently gained momentum worldwide. However, there is little information on the most suitable rearing conditions for growth, development and survivorship of these flies, which is a prerequisite for mass production technologies. We evaluated the physiological requirements for growth and reproduction of *H*. *illucens* on two diets [spent grains supplemented with brewers’ yeast (D1) and un-supplemented (D2)]. Development rates at nine constant temperatures (10–42°C) were fitted to temperature-dependent linear and non-linear day-degree models. Thereafter, life history table parameters were determined within a range of favourable temperatures. The thermal maximum (TM) estimates for larval, pre-pupal and pupal development using non-linear model ranged between 37.2 ± 0.3 and 44.0 ± 2.3°C. The non-linear and linear day-degree model estimations of lower developmental temperature threshold for larvae were 11.7 ± 0.9 and 12.3 ± 1.4°C for D1, and 10.4 ± 1.7 and 11.7 ± 3.0°C for D2, respectively. The estimated thermal constant of immature life stages development of BSF was higher for the larval stage (250±25 DD for D1 and 333±51 for D2) than the other stages evaluated. Final larval wet weight was higher on D1 compared to D2. The population growth rate was most favourable at 30-degree celsius (°C) with higher intrinsic rate of natural increase (*r*_*m*_ = 0.127 for D1 and 0.122 for D2) and shorter doubling time (5.5 days for D1 and 5.7 days for D2) compared to the other temperatures. These results are valuable for the optimization of commercial mass rearing procedures of BSF under various environmental conditions and prediction of population dynamics patterns using computer simulation models.

## Introduction

**Fig 1:**
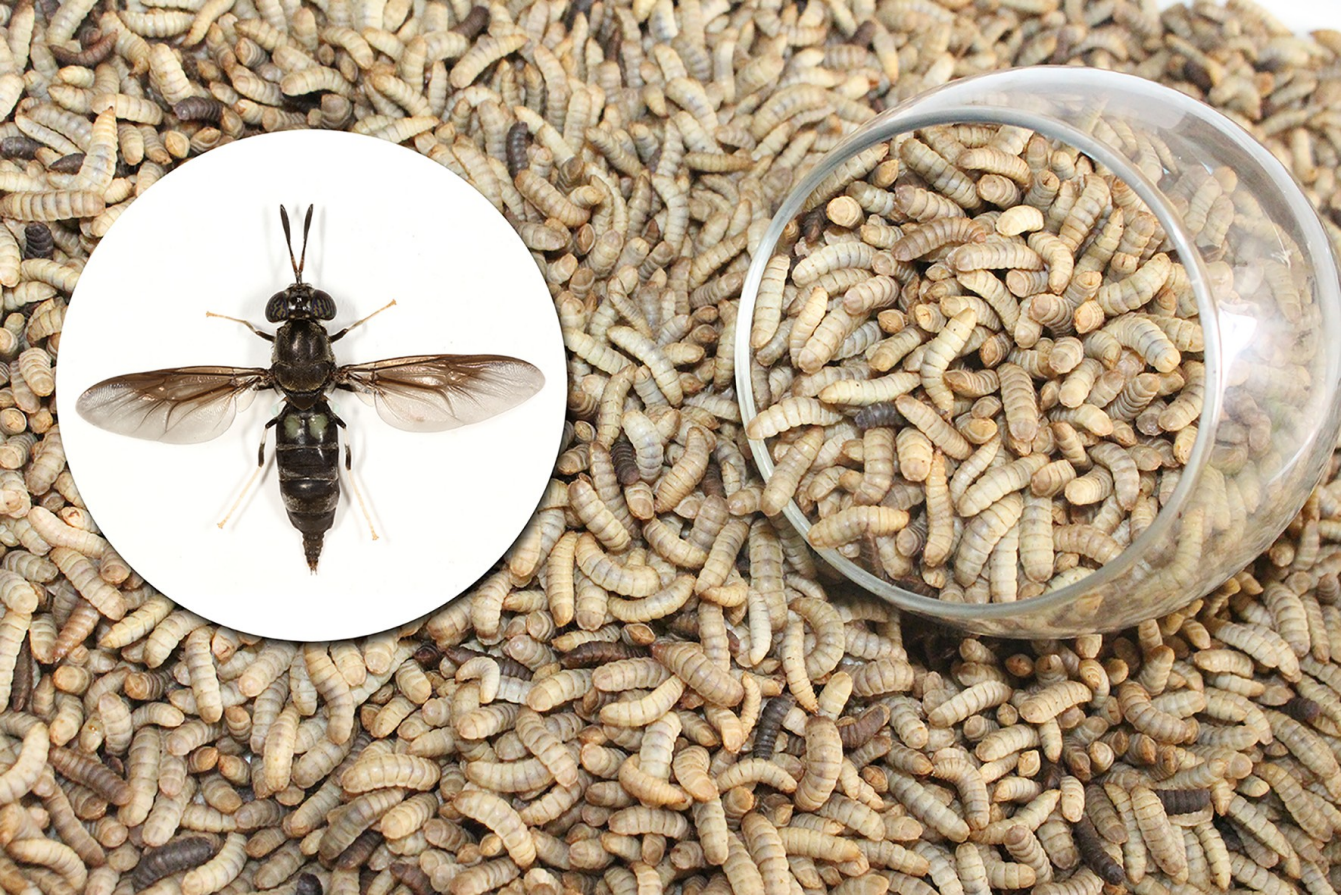
Illustration of black soldier fly (BSF) *Hermetia illucens* (Diptera: Stratiomyidae) and high-quality nutrient larval biomass.

The black soldier fly (BSF) *Hermetia illucens* L. (Diptera: Stratiomiydae) (Fig 1) is an indigenous saprophagous fly of the Neotropics region. However, the distributional range of these flies has widely changed over time to include the warmer parts of the world [1]. There has been substantial interest in the last decades to use these flies in organic waste management, given that their larvae are voracious eaters of organic waste (detritivores in compost heaps) [2–6]. The ability of BSF to convert waste into high-quality nutrient biomass has rapidly opened innovative economic prospects for municipal solid waste management in the different sectors. Also, the larvae of BSF after waste management are nutrient-rich consisting of an average of 42.1–43.2% crude protein, 33% fat and micronutrients [3,7–10], thus has been advocated as an appropriate alternative to fishmeal or soybean meal in poultry, pig and fish feeds [9,11,12] and provides opportunities for income generation [2,3].

Therefore, there has been increasing interest in developing novel methods of mass producing BSF as agents of organic waste management and composting as well as sustainable novel protein-rich ingredients in animal feeds. However, the paucity of scientific data and the reluctance of commercial producers to share detailed information impairs up-scaling BSF production technology among smallholder farmers [13]. As such knowledge on many important aspects related to mass production technologies of BSF remain poorly studied, especially temperature. Among these factors, temperature remains one of the most important factors [14,15] that considerably impacts behaviour, distribution, development rate, immature survival and reproduction, thus hampering the establishment of successfully rearing systems across the world [16].

To understand the dynamics and ecological system-specific BSF mass production strategies, data on temperature-driven population growth parameters of these insects become crucial, especially when dealing with small to medium-scale enterprises. Although information on development time, egg eclosion and adult emergence of BSF is reported for limited sets of temperatures [17–19], no information is available on temperature-driven effects on the full range of life history table parameters including mortality and reproduction at an extensive range of temperatures.

Several nonlinear models have been used [20, 21] to define the developmental and survivorship rates of different insects [22–25] over a varied array of temperatures but this has never been applied to BSF. Studies have shown that temperature significantly impacts the development and stage-specific immature life stages, thus directly impacting the quality and quantity of insects produced in rearing facilities [26–33]. Although, studies have shown that below and above the optimal temperature range of insect development, which is commonly limited by upper and lower developmental thresholds, survival does not occur but there are indications that this can vary depending on the insect-specific life stage or geographical origin of the insect species [34]. Thus, knowledge on developmental thermal requirements of insects will provide strong basis for comprehensive estimation of optimal response [35].

There is deficiency of information on temperature-driven development of BSF which is needed to maximize production. Thus, to enhance mass production technologies, additional studies are warranted on temperature requirements of BSF. In the present studies, we evaluated the stage-specific developmental time, survival, wet weight, pre-oviposition time, fecundity and adult longevity at nine constant temperatures between 10 to 42°C when BSF is reared on spent grains with or without supplementation with brewer’s yeast. The present work has implications for sustainable mass production of BSF as high-quality protein ingredient for animal feed.

## Materials and methods

### Origin of *H*. *illucens* population and colony maintenance

A stock culture of the wild-caught fly population was established following the methodology described by [36] and [37] with slight modifications. Egg-trapping of wild BSF was carried out in Kasarani, Nairobi County, Kenya (S 01° 13' 14.6''; E 036° 53' 44.5'', 1612 m above sea level). Chicken manure, rabbit manure, mixed fruit wastes and household food wastes were used as baiting materials for adult flies. The baiting materials were placed separately inside 6-L plastic buckets (22 cm height by 18 cm bottom diameter by 23 cm top diameter) designed with six openings (10 cm diameter each), 5 cm from the lid to facilitate entry of adult flies. The bait inside each container was maintained at approximately 70% moisture level. In cases where household food waste or mixed fruit waste were used, four–five holes were made at the bottom of the containers to allow excess fluid to drain out during the decomposition process. Three–five corrugated cardboard flutes (~10 cm length by 5 cm width) were attached vertically to the walls of the containers over the bait to serve as oviposition sites for adult flies. The lids of the containers were fastened to prevent desiccation or interference from heavy rainfall. Traps were labelled with date, location, time and GPS coordinates. Thereafter, the traps were hung on wooden or metallic stands (1 m above the ground) under shades around homesteads or close to garbage dump sites. We ensured that the wooden or metallic stands holding the traps were smeared with Tanglefoot^®^ (Tangle-trap) (Tanglefoot^®^ Company, Grand Rapids, MI) insect paste, which served as a barrier to stop intrusion by predators (ants, reptiles etc). These traps were checked regularly, and available egg clusters deposited by BSF in the cardboard flutes were harvested. The egg clusters were immediately transferred into other rearing containers holding a diet specifically formulated for the newly hatched neonates, hatching took approximately 4 days at 28 ± 1°C. Ten days after hatching the young larvae were transferred to bigger metal rearing trays containing wet brewer’s spent grains sourced from Kenya Breweries Limited, Nairobi, Kenya. The top surface of the rearing trays measured 76 cm length by 27.5 cm width x 10 cm height, while the bottom of the tray measured 52 cm length by 27.5 cm width, which allowed both edges of the tray to be inclined at a 35-degree angle. In rearing facility, conditions were maintained at 28 ± 1°C, 65 ± 5% RH and L12:D12 photoperiod. User friendly thermo-hygrometers (TH-812E) were maintained in each rearing room to allow us track slight changes in temperature and RH.

Pre-pupal stages harvested from the rearing trays were kept in 4-L transparent rectangular plastic containers (21x14x15 cm) (Kenpoly Manufacturer Ltd., Nairobi, Kenya) containing moist wood shavings (sawdust) until eclosion. On the lid of each container, an opening (14.5x8.3 cm) was introduced and screened with fine netting organza material. Emerging flies were transferred to an outdoor cage with a base of (1x1x1.8 m). The flies were provided *ad libitum* 60% sugar solution on cotton wool or soaked pumice granules in 2-L rectangular transparent plastic containers (21x14x7 cm) (Kenpoly Manufacturers Ltd., Nairobi, Kenya). Flies were fed for 7 days until sexual maturity, when body colorations were fully expressed. Some of the flies (15 males and 15 females) were rendered inactive by freezing them (-20°C) for 20 min and later preserved in 90% alcohol for taxonomic identification based on morphological features [38, 39]. Thereafter, the preserved specimens were identified at the Biosystematics unit of *icipe*, which also holds reference materials of the specimens. BSF has been in culture for ~2 years. In addition, BSF was kept at high numbers (2000–2500 adults per cage) to avoid inbreeding depression while also avoiding stressful crowding effects [40].

### Sources of experimental substrates and diet formulation

Before the commencement of the experiment, fresh brewer's spent grains (BSGs) (malt/corn starch) and brewer’s yeast, both by-products of brewing Tusker beer were sourced from the Kenya Breweries Limited, Nairobi, Kenya. The fresh BSGs were subsequently dried using moving dry air at 28.0 ± 2°C (using Xpelair*®* heater: WH30, 3KW Wall Fan Heater, United Kingdom) for two days (48 h). Afterwards, the semi-dried products were maintained in the oven for 3-days (72 h) at 60°C to dry properly to achieve approximately 90% DM (~10% moisture). The dried BSGs were later passed through the Münch hammer mill (Münch, Wuppertal, Germany) to reduce the materials to 3 –mm particle size, suitable for incorporation into BSF diet when needed.

Two experimental diets were then formulated for the BSF larvae, which consisted of diet one (D1) (50 g BSGs mixed in 90 ml of brewer’s yeast) and diet two (D2) (50 g BSGs mixed in 80 ml of water (Control). Each diet was hydrated to approximately 70.0±2% moisture by weight according to the protocol described by Cammack and Tomberlin [41] and confirmed using a moisture sensor with two 12-cm-long probes (HydroSense^TM^ CS620, Campbell Scientific, Inc., Logan, UT, USA). Five replicates were conducted for each experimental diet fed *ad libitum* until the late larval stage.

### Temperature effect on the development and survivorship rate of *H*. *illucens* life stages

Thermostatically controlled incubators (MIR-554-PE, Sanyo/Panasonic cooled incubators, Japan) were used to conduct the experiments set at one of the nine constant temperatures conditions [(10, 15, 20, 25, 30, 35, 37, 40 or 42°C (± 0.03°C)]. The relative humidity in each incubator was maintained at 70 ± 2.0% and photoperiod of 12:12 L: D. In each incubator, the effect of temperature on the developmental time and survival of the different life stages were assessed. EasyLog USB data loggers (EL-USB-2, RH/Temp data logger; MicroDAQ.com, Ltd. USA, 603-746-5524) were placed inside each incubator, which recorded the inside temperature at 15 minutes interval.

At the start of the experiments, 18 well-ventilated transparent Perspex cages (40x40x70 cm) (nine cages for each diet) were prepared and each provided with 350, 7-days-old mated females at each temperature treatment. Eggs of BSF were obtained by providing the flies with oviposition media, that consisted of corrugated cardboard with flutes (cut into sizes as described above). The cardboards were attached to the wall of the cages near the baiting materials (described above) and on top of the substrates. Freshly laid egg clusters were obtained from the stock colony at regular intervals of 1 hour after the eggs were laid.

#### Egg

Camel hair brush with a fine tip was used to collected 300 eggs (~1 hr old) randomly, which were later counted and carefully transferred unto sterilized Petri dishes (150 x 25 x 20 mm). The Petri dishes containing the eggs were transferred to the nine incubators described above. The experimental setup with the eggs were monitored at regular interval of 6 hours daily until they hatched. All eggs eclosion at each temperature regime was recorded with the help of entomological tweezers under the microscope (Leica MZ 125 Microscope; Leica Microsystems Switzerland Limited). The stereomicroscope used for counting the emerged neonates was fitted with Toshiba 3CCD camera and an auto-montage software (Syncroscopy, Synoptics Group, Cambridge, UK) at 25X magnification to ensure no damage was observed. Time until hatching of larvae and percentage egg hatch was determined. The experiments were replicated five times per temperature treatment.

#### Larva

Per temperature treatment, 300 newly hatched larval BSF (~1 hr old) were randomly obtained from the clusters of hatched eggs maintained at each experimental temperature and transferred onto square (5 cm^2^) sterile filter paper. The square filter papers with the young larvae BSF larvae were transferred onto a 50 g of formulated diets of either diet 1 or 2 (D1 & D2) placed in 2-L rectangular plastic rearing containers. The plastic containers containing the neonates were then maintained in respective thermostatically controlled incubators. The opening made on the lids of the containers were covered with netting of 1.3x1.3 mm mesh size to allow for sufficient ventilation. The larvae were then fed *ad libitum*, until pre-pupal stage. Fifty 5^th^ instar larvae of similar age were randomly selected from each diet in the different temperature treatments and separated into ten replicate groups of five and weighed to determine the wet weight. Stage-specific developmental time and survival was recorded for each temperature treatment. For each experiment in the different temperature regimes, five replications were achieved.

#### Pre-pupa

A total of 300 newly formed 1-hour old pre-pupae were selected randomly from the experimental culture maintained on each diet at each temperature treatment. The pre-pupae used for the experiment were maintained individually in small plastic containers with a top and bottom diameter of 5 and 4 cm, respectively and a height of 4 cm. The containers were provided with breathable lids. Each container had 2.5 cm-deep moist sawdust, which served as pupation substrates. The containers used for the experiment were monitored daily for developmental and survival rate of the pupal stage. Each experiment was replicated five times.

#### Pupa

From experimental cultures maintained on each diet at each temperature, 300 pupae (~ 1 hr old) were randomly selected and transferred individually into plastic containers (4 x 5 cm diameter x 4 cm height) with 2.5 cm- layer of moist sterile sawdust for emergence. Pupae that failed to emerge within the anticipated period were allowed for an addition 1-month period before pronouncing them dead. Emerged adults were recorded by sex, and their wet adult weight (50 individuals) recorded. Each experiment was replicated five times for each diet and temperature treatment.

### Temperature effect on adult *H*. *illucens* fecundity, oviposition and longevity

Upon emergence, one female and one male adult *H*. *illucens* (~1 hr old) were paired. The individual BSF adult pairs were placed in well ventilated Perspex cages (15 x 15 x 10 cm). In each cage, the paired flies were provided sugar water on soaked cotton balls. Thereafter, the flies were provided with two corrugated cardboard flutes stocked on the walls of the container near the substrate. The corrugated cardboards were maintained in the cages throughout the lifespan of the flies. Egg cluster(s) produced daily were checked and counted, while the longevity of individual flies was also recorded. In each egg cluster collected, total of number of eggs laid per female throughout their life span was counted and recorded. Thirty pairs of *H*. *illucens* were monitored for each temperature treatment and diet. Pre-oviposition duration was calculated based on the days required for a newly emerged female BSF to start ovipositing. Adult longevity was calculated based on the length of time lived by adult fly from the date of emergence until death. The fecundity was considered as the eggs laid per female throughout their lifetime.

In addition, a group of 100 newly emerged adult female flies and 100 males (~1 hr old) were kept in groups in Perspex cages (30x30x30 cm) subjected at different temperature regimes. The longevity and percentage survival of flies were recorded.

### Life history parameters

The net reproductive rate (*R*_*o*_), intrinsic rate of natural increase (*r*_*m*_), generation time (*G*), and doubling time (*DT*) were estimated using the method described by Carey [42] using the modified spreadsheet of [43]. The net reproductive rate (*R*_*o*_), which is an indication of the number of offspring that an individual female fly can produce during its life span was calculated as below.

$$R_{o}=\sum_{x=0}^{w}l_{x}m_{x}$$

The maximum population growth, intrinsic rate of natural increase (*r*_*m*_) was assessed using the iterative bisection approach from the Euler-Lotka equation with age indexed starting from zero. Life table with data on the *r*_*m*_ at different temperature regimes provide insight into the characteristic life patterns of BSF.

$$\sum_{x=0}^{w}e^{-r(x+1)}l_{x}m_{x}=1$$

The finite rate of increase (λ), which represents overall female offspring per female per day, was calculated using the formula below,

$$\lambda =e^{rm}$$

The mean generation time (T), which is defined as the length of time that a population requires to increase to *R*_*o*_-fold of its population size at the stable age-stage distribution was estimated using the formula

$$T=\frac{{lnR}_{o}}{r_{m}}$$

The gross reproductive rate (GRR) was calculated as

$$GRR=\sum_{x=0}^{w}m_{x}$$

The doubling time, is defined as the overall days required by a population to double and was calculated as shown below:

$$DT=\frac{\ln\left(2\right)}{r_{m}}$$

where *l*_*x*_ is the female survival rate from egg to age *x*, *w* is the oldest surviving age, *x* is the age class in days and *m*_*x*_ is mean female progeny per female occurring during age *x*. The means, variances and standard errors of the different parameters were calculated via the bootstrap procedure [44].

### Temperature-dependent models

For temperature dependent models, both linear [45] and nonlinear model were fitted to developmental rate stage-specific data of the insect. The linear model expressed below evaluated the relationship between *H*. *illucens* developmental rates and temperatures.

r(T)=a+bT

In the model, *T* is the ambient temperature (°C), *r* is an indication of the development rate [1/developmental duration presented in days], while the intercept (*a*) and slope (*b*) are model parameters. The *T*_*min*_ and standard error were calculated using the following equations [45]:

$$T_{min}=\frac{-a}{b}$$

$$SE_{T_{min}}=\frac{y_{m}}{b}\sqrt{\frac{S^{2}}{N\times y_{m}{}^{2}}+{\left[\frac{SE_{b}}{b}\right]}^{2}}$$

where *y*_*m*_ is the average value of the developmental rate, *b* is estimated slope of fitted line, *S*^2^ is the residual mean square of the linear model, and *N* is the sample size [46]. The total thermal energy (heat units) necessary to complete development, which is considered to be above the low temperature development thresholds (LTDT) and referred to as the thermal constant *K*, *is represented* in degree-days (DDs). The value of *K* and its standard error (*SE*_*k*_) were calculated using the following equation [45]:

$$K=\frac{1}{b}$$

$${SE}_{k}=\frac{{SE}_{b}}{b^{2}}$$

Many empirical non-linear models fitted to developmental rate stage-specific data has been used to determine minimum temperature thresholds (*T*_*min*_), optimal temperature thresholds (*T*_*opt*_) and upper temperature thresholds (*T*_*max*_). Optimum temperature (*T*_*opt*_) is defined as the temperature when developmental rate is observed to be maximal, while *T*_*max*_ is referred to threshold temperatures above which temperatures are lethal. Between the different non-linear models evaluated, Brière 1 model provided a best explanation of temperature effect on the development of *H*. *illucens* life stages in comparison to other models tested. Brière 1 model is expressed as below:

$$r(T)=n*T*(T-T_{min})*\sqrt{T_{max}-T}$$

Here, *r* is considered as the developmental rate, derived as function of temperature *T*, *n* being an empirical constant, *T*_*min*_ the lower development temperature threshold, and *T*_*max*_ the lethal upper temperature threshold. The optimal temperature (*T*_*opt*_) of *H*. *illucens* development rate was estimated using the following equation [21]:

$$T_{opt}=\frac{2mT_{max}+(m+1)T_{min}+\sqrt{{({4m}^{2}T}_{max}^{2}+{{\left(m+1\right)}^{2}T}_{min}^{2}-{{4m}^{2}T}_{min}T_{max})}}{4m+2}$$

where *m* = 2 [21].

### Statistical analysis

A two-way analysis of variance (ANOVA) was used to analyse the data on development time and survival of immature life stages, adult longevity, fecundity and pre-oviposition period (dependent variables) to evaluate the effect of temperature and rearing substrate and their interaction (independent factors). Percentage of survival and average wet weight for the different life stages of BSF at different temperatures and rearing substrates were also analysed using two-way ANOVA. In the event of a significant *F* test (*P* < 0.05), the Student Newman Keuls (SNK) test was used to compare means. Prior to analysis of variance, all proportional data (percentage survival and emergence) were transformed using angular transformation to stabilise variance. All statistical analyses were performed using R version 3.4.1 (R Core Team, 2017) [47].

## Results

### Temperature effect on developmental and survival rate of *H*. *illucens* immature life stages and adults

**Fig 2:**
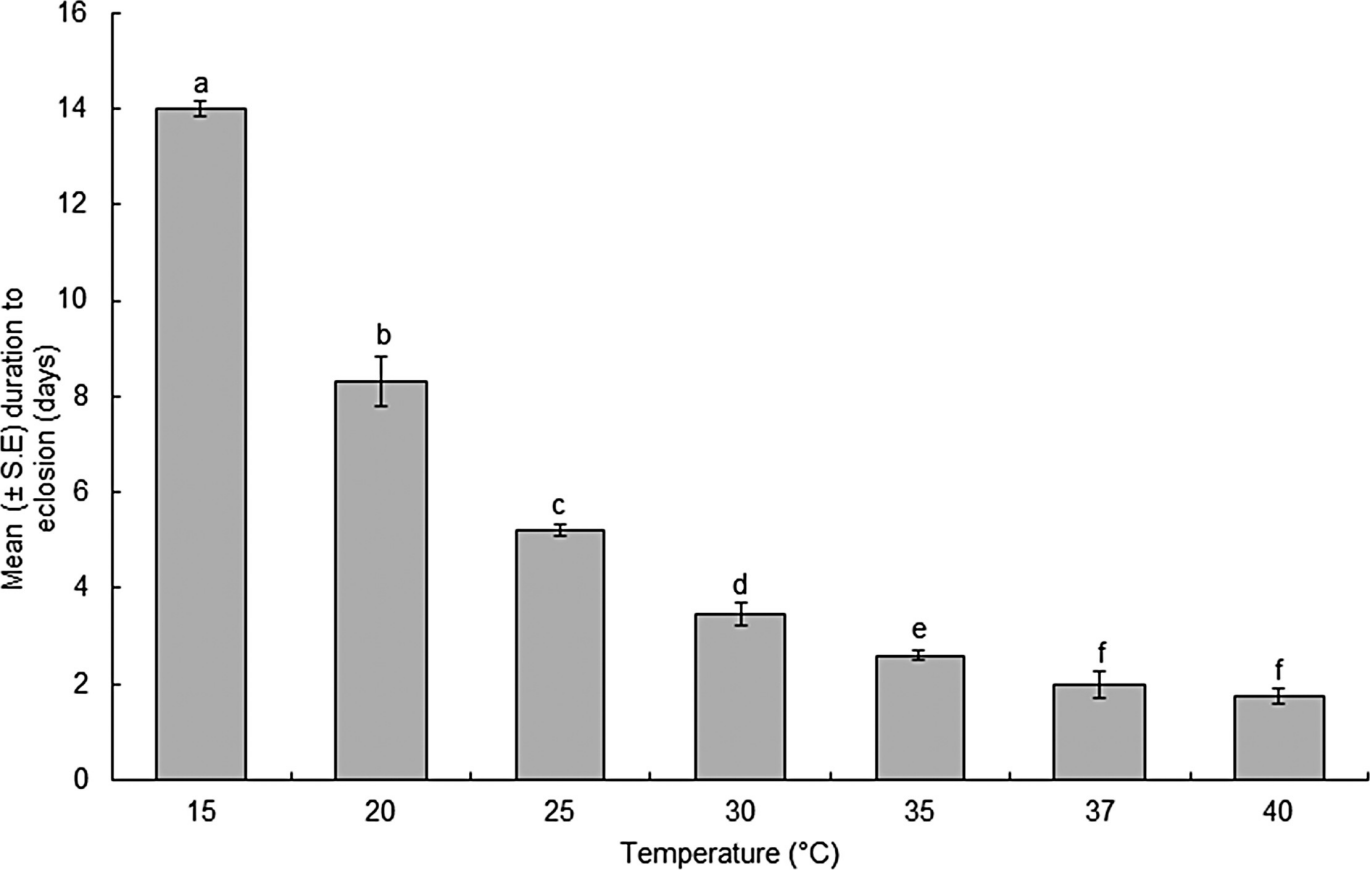
Duration of black soldier fly egg eclosion at different temperatures. Means with different lower-case letters are significantly different (*P* < 0.05, SNK test).

The time to egg eclosion differed significantly (F = 117.3; df = 6, 19; *P* < 0.0001) among the different temperatures (Fig 2). The eclosion time of the eggs incubated at 15°C was 14-days, compared to those incubated at 35°C, which eclosed in 2.60 days. After monitoring the egg experimental set-up for over 40 days, eggs at 10 and 42°C were observed to have completely collapsed and deemed not viable.

**Fig 3:**
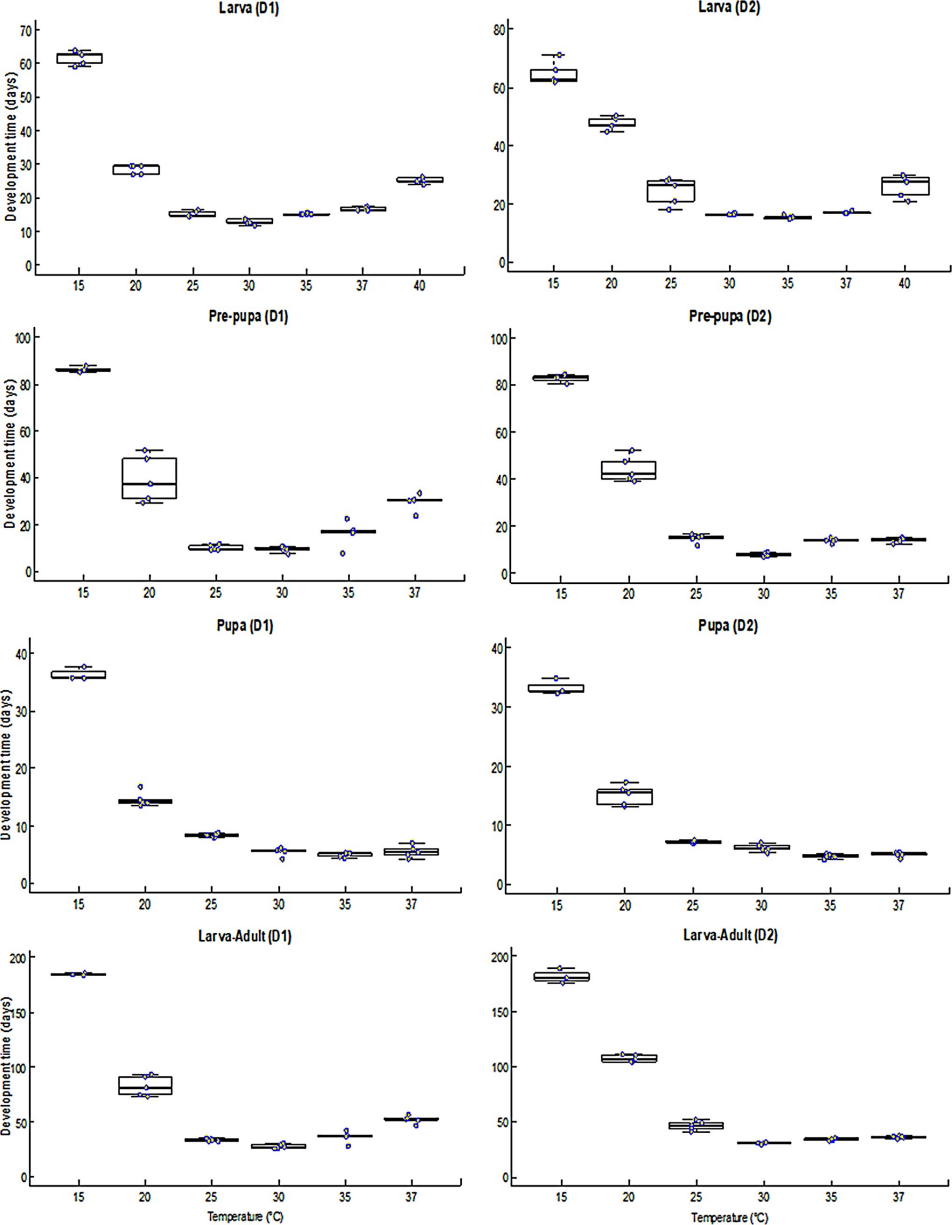
Development time of different life stages of black soldier fly fed on two diets at constant temperatures. The middle quartile or the median (line that divides the box into two parts) marks midpoint of the data. Middle box represents 50% of the scores for each treatment and the middle 50% values fall within the inter-quartile range.

Larval development time differed significantly among temperatures (F = 843.1; df = 6,28; *P* < 0.0001 for D1 and F = 153.5; df = 6, 28; *P* < 0.0001 for D2) and between rearing substrates (F = 114.1; DF = 1, 56; *P* < 0.0001). There was significant interaction (F = 20.2; df = 6, 56; *P* < 0.0001) between the effect of temperature and rearing substrate on larval developmental time. Larval developmental time on D1 ranged between 12.8 ± 0.34 days at 30°C and 61.6 ± 0.91 days at 15°C (Fig 3). The longest larval developmental time was recorded at 15°C (65 days), while the shortest developmental time was at 30°C (13 days) and 35°C (16 days) when reared on D1 and D2, respectively (Fig 3). Larvae reared on D1 and D2 had significantly different developmental time at 20, 25 and 30°C but similar developmental time at 15, 35, 37 and 40°C (Fig 3).

The pre-pupal stage developed from larvae fed on D1 and D2 failed to complete development to pupa at 40°C. The pre-pupal development times differed significantly among temperatures tested (F = 61.22; df = 5, 22; *P* < 0.0001 for D1 and F = 349.3; df = 5, 22; *P* < 0.0001 for D2) and between the different diets (F = 4.743; df = 1, 44; *P* = 0.0350). The interaction (F = 12.35; df = 5, 44; *P* < 0.0001) between the effect of temperature and diet was significant. Pre-pupae from larvae reared on D1, showed significant variation in developmental times at 15, 20, 25, 30 and 35°C, but similar values at 20 and 37°C (Fig 3). The longest pre-pupal developmental time was 86 and 83 days at 15°C for D1 and D2, respectively, while the developmental time was shortest at 30°C (10 and 8 days for D1 and D2, respectively) (Fig 3).

The development time of pupae obtained from larvae fed on D1 and D2, displayed significant variation between the different temperatures (F = 175.4; df = 5, 22; *P* < 0.0001 and F = 304.5; df = 5, 22; *P* < 0.0001, respectively). The effect of larval rearing diets (D1 and D2) on pupal developmental time did not differ significantly (F = 2.002; df = 1, 44; *P* = 0.1640) across the different temperature. No significant interaction was observed between the effect of temperature and diet on pupal development (F = 2.272; df = 5, 44; *P* = 0.0642).

Overall developmental duration from larva to flies showed a significant difference among the temperature regimes (F = 209.3; df = 5, 22; *P* < 0.0001 and F = 885.4; df = 5, 22; *P* < 0.0001 when reared on D1 and D2, respectively) and between diets (F = 5.25; df = 1, 44; *P* = 0.0274). The effect of temperature and diet on developmental time showed a significant interaction (F = 26.54; df = 5, 44; *P* < 0.0001). Using diet 1 (D1), the developmental time from larva to adult ranged between 28 days at 30°C to 184 days at 15°C, whereas those reared on D2, completed development to adult in 31 days at 30°C and 181 days at 15°C (Fig 3).

### Most favorable temperature range of black soldier fly

The stage-specific survival of BSF at nine constant temperatures is presented in Table 1. Egg viability was extremely low (below 11%) at 15, 37 and 40°C compared to the other temperatures. The highest percentage of egg eclosion was recorded at 30°C (80%) and 35°C (75%) (Table 1).

**Table 1:** Percentage survival (mean (±SE) of immature life stages of black soldier fly at constant temperatures.

Temperature (°C)	Egg	Larva		Pre-pupa		Pupa	
		D1	D2	D1	D2	D1	D2
10	-	-	-	-	-	-	-
15	10.6 ± 2.1c	82.8 ± 8.8aA	87.0 ± 3.5aA	58.7 ± 1.3bA	65.3 ± 6.4abA	49.3 ± 7.1bA	62.0 ± 3.1bA
20	59 ± 10.6b	74.6 ± 5.7aA	82.4 ± 5.4aA	68.4 ± 5.2abA	61.0 ± 2.7abA	61.6 ± 4.5abA	59.4 ± 1.9bcA
25	59.8 ± 8.5b	93.0 ± 1.4aB	58.0 ± 5.0bA	83.1 ± 2.9aB	54.4 ± 5.0bA	67.4 ± 4.0abB	46.3 ± 2.5cA
30	80.0 ± 5.6a	92.6 ± 2.4aA	90.4 ± 0.7aA	82.2 ± 4.2aA	77.2 ± 4.0aA	77.1 ± 5.9aA	74.8 ± 4.2aA
35	74.8 ± 14.7a	90.8 ± 1.7aA	92.2 ± 3.5aA	75.2 ± 3.1aA	79.0 ± 4.5aA	65.6 ± 3.3abA	54.1 ± 5.9bcA
37	9.8 ± 3.1c	89.4 ± 6.3aA	84.0 ± 1.5aA	24.1 ± 3.0cB	63.6 ± 6.8abA	5.4 ± 1.0cB	19.6 ± 2.4dA
40	9.4 ± 2.1c	34 ± 15.0bA	27.6 ± 7.7cA	-	-	-	-
42	-	-	-	-	-	-	-

D1 = diet 1, D2 = diet 2. For eggs, means within the same column followed by different lower-case letter are significantly different (*P* < 0.05, SNK Test). For the larval, pre-pupal and pupal stages, means within the same column followed by different lower-case letter are significantly different (*P* < 0.05, SNK Test) for D1 or D2. For the larval, pre-pupal and pupal stages, means within the same row for each life stage followed by the same upper-case letter are not significantly different (*P* < 0.05, T-test) for D1 or D2. (-) no survival was observed.

Percentage larval survival differed significantly (F = 7.68; df = 6, 28; *P* < 0.0001 for D1 and F = 19.79; df = 6, 28; *P* < 0.0001 for D2) among the different temperature treatments. The interaction between the effects of temperature and diet was significant (F = 2.60; df = 6, 56; *P* = 0.0217). However, survival rate was comparable at 15, 20, 25, 30, 35 and 37°C, whereas it was lower at 40°C (Table 1). For D2, larval survival rate was high at 35°C (92%) and 30°C (90%) and low at 40°C (28%).

Pre-pupal survival was significantly influenced by temperature when larvae were reared on D1 (F = 26.58; df = 5, 22; *P* < 0.0001) and D2 (F = 4.08; df = 5, 22; *P* = 0.0090). There was a significant interaction (F = 11.91; df = 5, 44; *P* < 0.0001) between temperature and rearing diets. For D1, the highest pre-pupal survival rate was recorded at 25°C (83%) and 30°C (82%), while the lowest value was recorded at 37°C (24%). For D2, the highest percentage of survivorship was observed at 35°C (79%) and 30°C (77%), whereas at 25°C the survival rate was the lowest (54%). However, the survival rate across the different temperature regimes was similar, except at 25°C.

The pupal survival rate differed significantly between temperatures when the larvae were reared on D1 (F = 42.28 = df = 5, 22; *P* < 0.0001) or D2 (F = 25.96; df = 5, 22; *P* < 0.0001). The interaction between the effect of rearing diet and temperature on pupal survival was significant (F = 6.67; df = 5, 44; *P* < 0.0001). The percentage of survival recorded for the pupal stages were observed to decrease from 77% at 30°C to 5% at 37°C for D1 and from 75% at 30°C to 20% at 37°C for D2 (Table 1).

### Longevity and reproduction of black soldier fly at different temperature regimes

**Fig 4:**
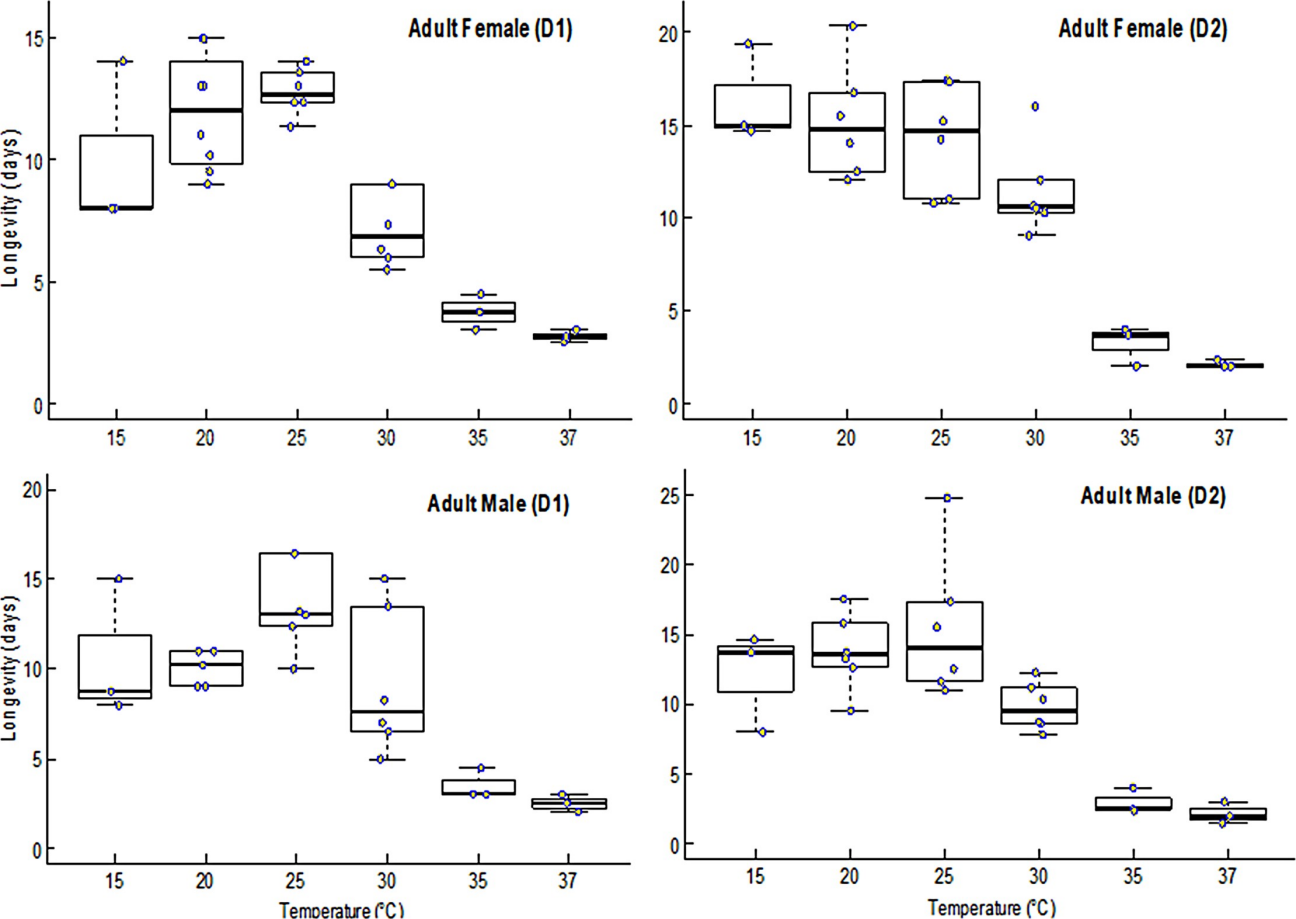
Boxplots showing adult black soldier fly female and male longevity at constant temperatures. Middle quartile (line that divides the box into two parts) shows midpoint of the data. Middle box represents 50% of the scores for each treatment and the middle 50% values fall within the inter-quartile range.

**Fig 5:**
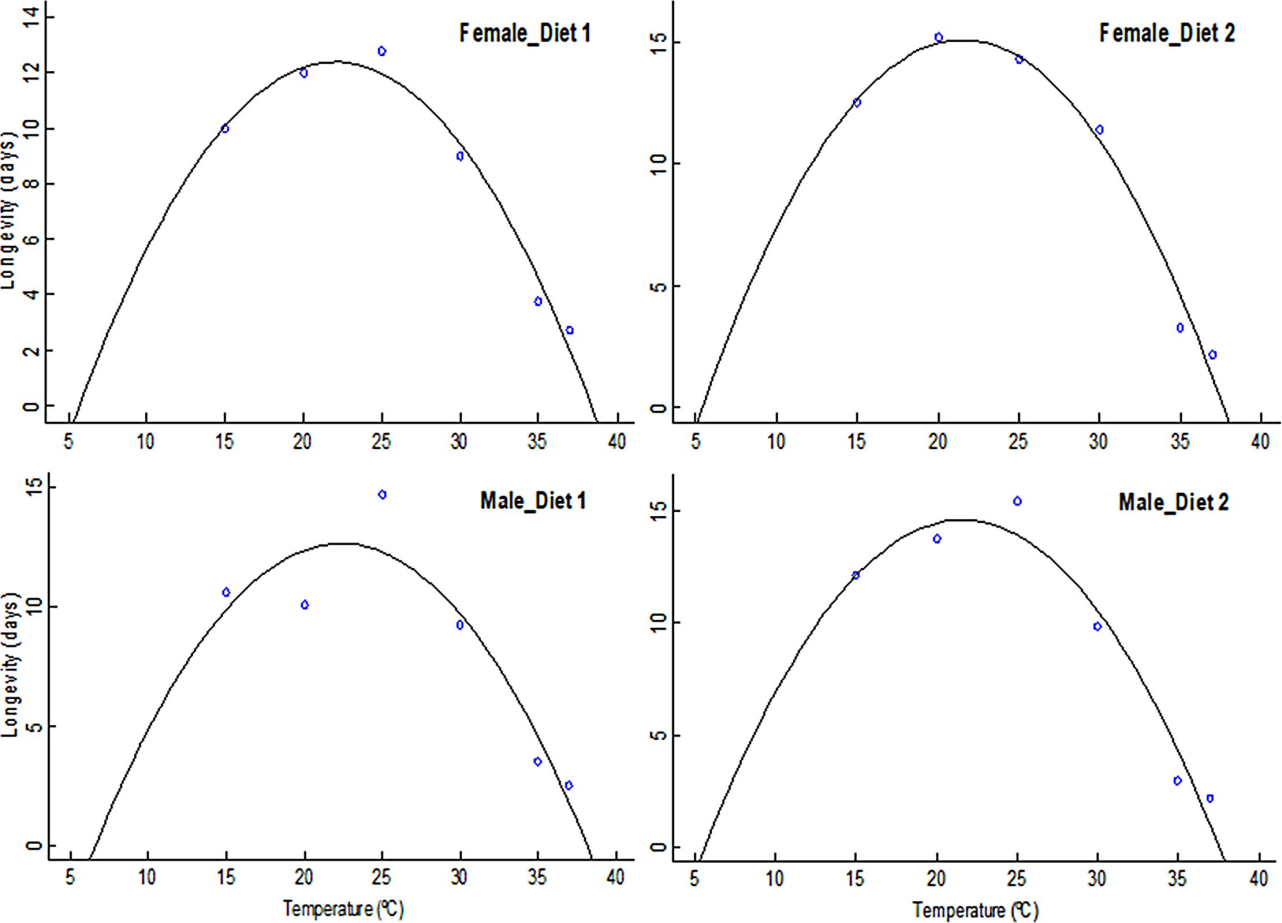
Quadratic model curves describing longevity of adult female and male black soldier flies at constant temperatures. Quadratic: *n(T)* = *a + bT + cT*^*2*^; *n(T)* represents the longevity function at temperature T (°C); *a*, *b* and *c* are empirical parameters of the model.

**Fig 6:**
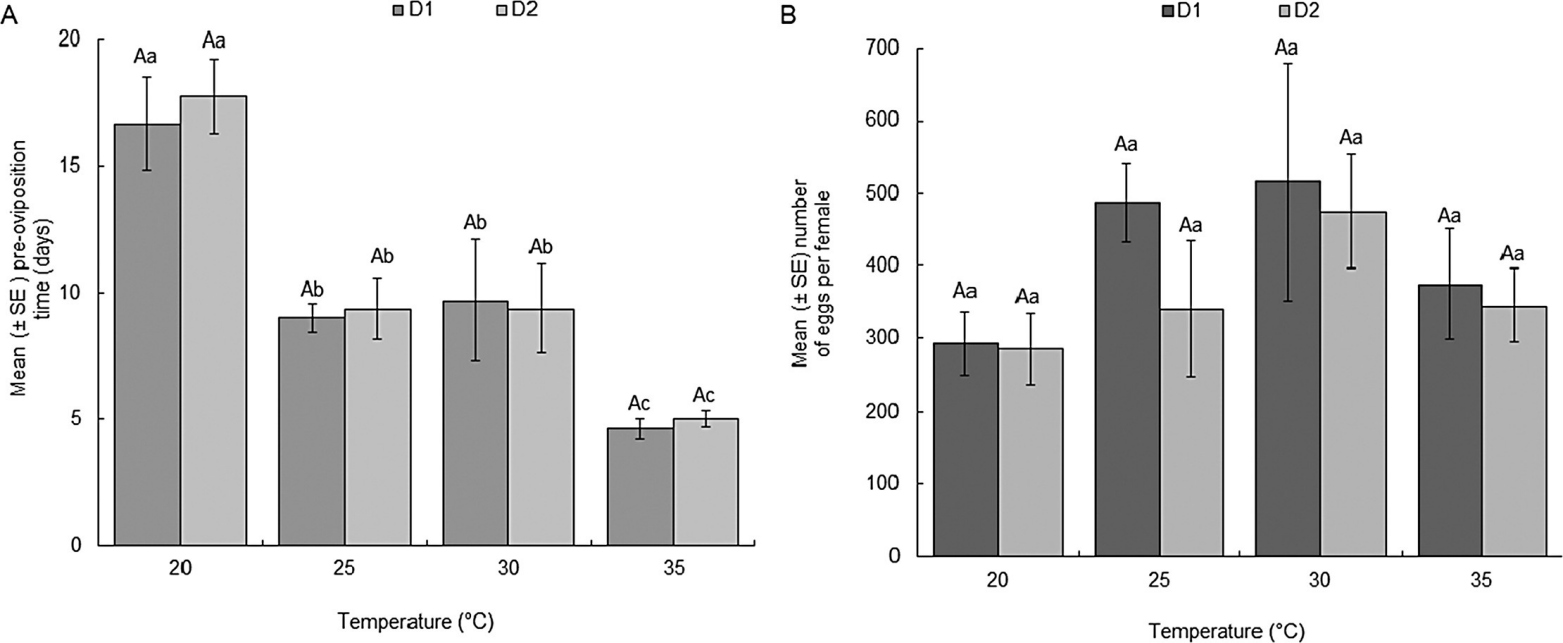
Mean number of days before oviposition of egg clutch by female black soldier fly (pre-oviposition period) (A) and mean number of eggs laid per female during her lifespan (B) at constant temperatures.

The longevity of adult BSF was significantly affected by temperature for both females (F = 52.48; df = 5, 29; *P* < 0.0001) and males (F = 53.27; df = 5, 29; *P* < 0.0001). Fig 4 indicates that adult flies live longer at intermediate temperatures than at upper extreme temperatures as illustrated by the quadratic model fitted to longevity (Fig 5) for both sexes when their larvae were reared on D1 or D2. Average pre-oviposition period was observed to vary significantly across the different temperatures being longest at 20°C (16 days) and shortest at 35°C (5 days) when larvae were reared on D1 and D2 (Fig 6). Fecundity was significantly affected by temperature, especially at the lower (15°C) and upper (37°C) temperatures evaluated. The highest fecundity of BSF was observed at 30°C (516 and 475 eggs when flies were reared on D1 and D2, respectively) (Fig 6).

### Body weight of black Soldier fly life stages reared on two diets at constant temperatures

**Fig 7:**
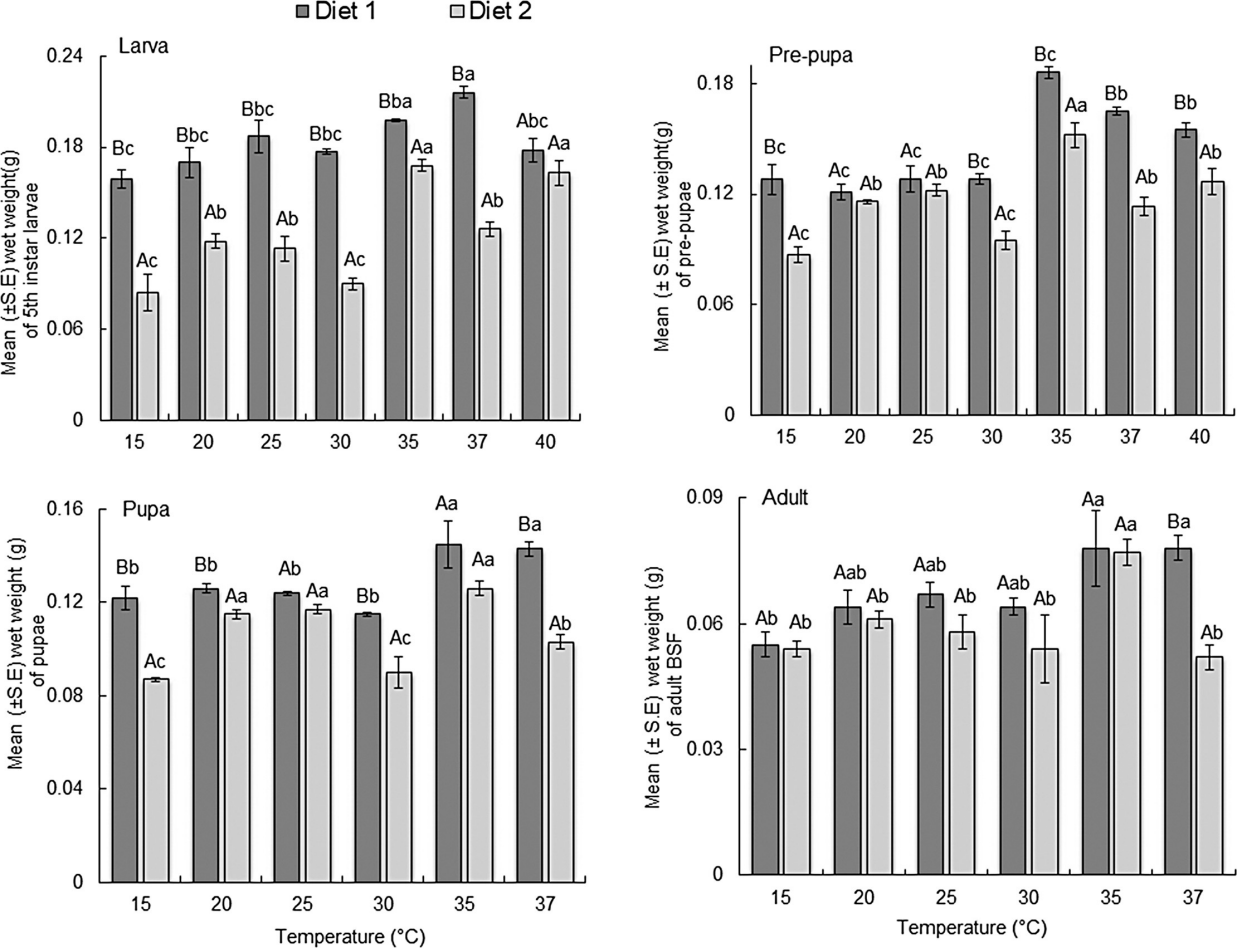
Mean wet weight of black soldier life stages reared on two diets at constant temperatures. Means within the same treatment with the same lowercase letter are not significantly different. Means within the same temperature regime with the same uppercase letter are not significantly different (*P* < 0.05, SNK).

The mean wet body weight for the different life stages of BSF reared on two diets are presented in Fig 7. The estimated mean wet weight was higher for all BSF life stages reared on D1 compared to D2 across the different temperature regimes. Body weight of 5^th^ larval instar varied significantly among the different temperature regimes (F = 6.26; df = 6, 26; *P* < 0.0001 for D1 and F = 25.8; df = 6, 26; *P* < 0.0001 for D2) and between rearing diets (F = 267.81; df = 1, 52; *P* < 0.0001). The interactions (F = 9.52; df = 6, 52; *P* < 0.0001) between the temperature and diet was found to be significant. Mean larval weight was highest (0.216 g) at 37°C and lowest (0.159 g) at 15°C when reared on D1 while on D2, mean larval weight was highest (0.168 g) at 35°C and lowest (0.084 g) at 15°C.

Pre-pupal weight differed significantly across the temperature treatments (F = 31.59; df = 6, 26; *P* < 0.0001 for D1 and F = 15.94; df = 6, 26; *P* < 0.0001). A significant interaction was observed (F = 6.78; df = 6, 52; *P* < 0.0001) between temperature and diet for pre-pupal weight. The highest mean pre-pupal weight when the larvae were fed on D1 (0.186 g) or D2 (0.152 g) was recorded at 35°C and the lowest at 20°C (0.121 g) and 15°C (0.087 g) for D1 and D2, respectively.

Pupal weight was significantly different between temperature regimes (F = 6.63; df = 5, 22; *P* < 0.0001 for D1 and F = 17.22; df = 5, 22; *P* < 0.0001 for D2) and between the rearing diets (F = 77.28; df = 1, 44; *P* < 0.0001). There was a significant interaction (F = 4.56; df = 5, 44; *P* = 0.0020) between the effects of temperature and rearing diet. The highest mean pupal weight was recorded at 35°C for either D1 (0.145 g) or D2 (0.126 g) while the lowest weight was recorded at 15°C for either D1 (0.122 g) or D2 (0.087 g).

Adult weight varied across the temperature treatments for each diet (F = 113.2; df = 6, 26; *P* < 0.0001 for D1 and F = 245.2; df = 6, 26; *P* < 0.0001). The highest mean adult body weight was recorded at 35°C for either D1 (0.078 g) or D2 (0.077 g).

### Temperature-dependent developmental models of black soldier fly

**Fig 8:**
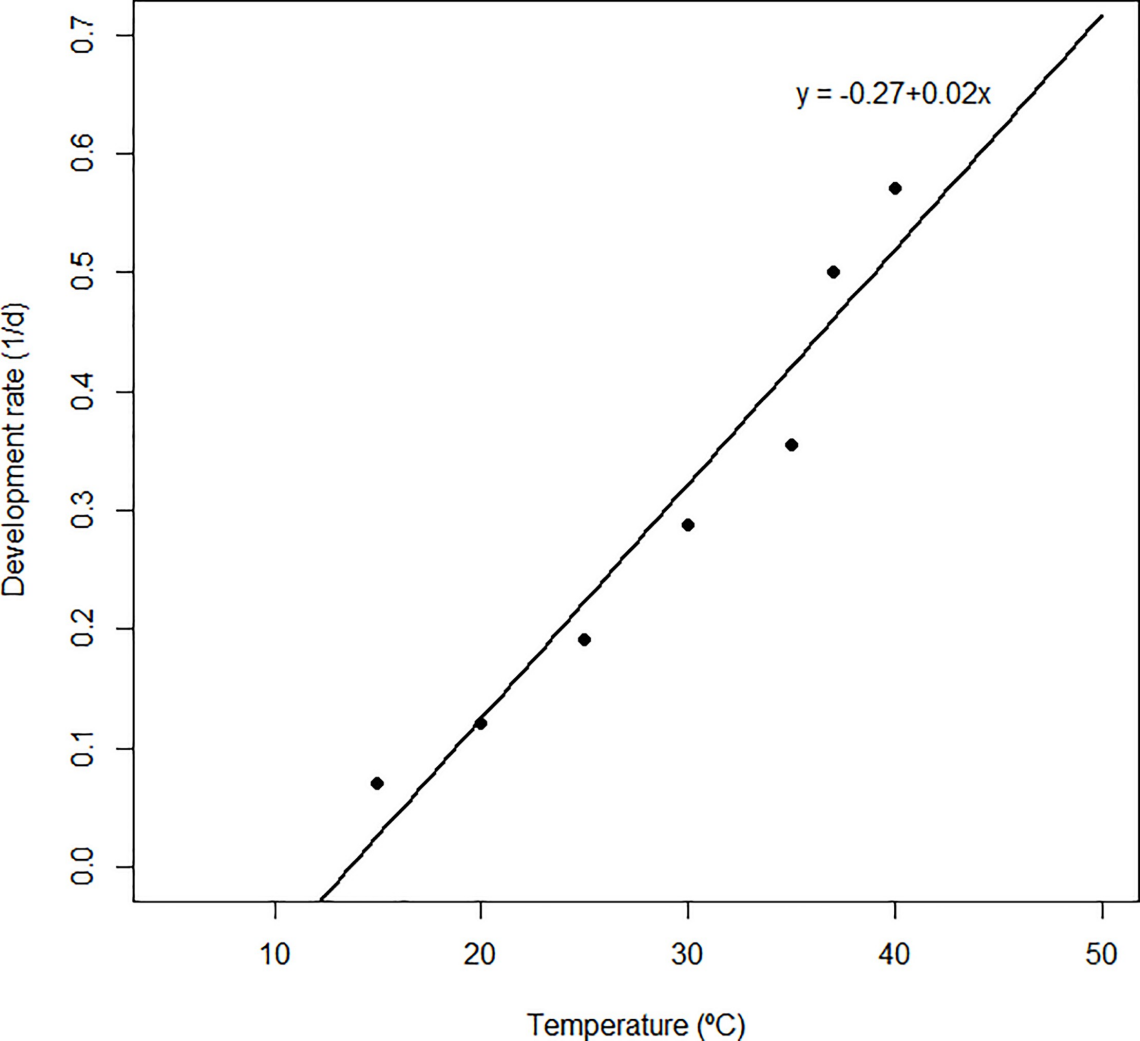
Linear model fitted to observed values of development rate of black soldier fly eggs at constant temperatures.

Table 2 for BSF eggs and Table 3 for immature life stages present the parameter estimates obtained from the non-linear Brière-1 model and linear models fitted to developmental rate. The fitted models for developmental rates (1/d) versus temperature for all the different life stages are presented in Figs 8 (egg) and 9 (larval, prepupal and pupal stages). The lower temperature threshold (*T*_*min*_) for larval, pre-pupal and pupal stages estimated using Brière-1 model were lower as compared to estimates of the linear regression model on both diets. Using the linear model, *T*_*min*_ of BSF eggs was estimated at 13.6°C. For the larvae, pre-pupae and pupae, *T*_*min*_ was estimated as 12.3, 13.3 and 13.3°C, respectively, for D1, and 11.7, 14.6 and 12.2°C, respectively, for D2 (Table 3), which were all similar to that of Brière-1 model estimates. The optimal temperature threshold for larval, pre-pupal and pupal developmental stages reared on D1 were estimated as 31.3–36.0°C, and 32.3–36.4°C for D2. The lethal upper temperature thresholds were estimated to range from 37.2–44.0°C for the different immature life stages (Table 2 and Table 3). The BSF egg required 69.24 degree-days (DD) for the successful completion of eclosion, whereas the larval, pre-pupal and pupal stages required 250.2, 142.9 and 142.9 DD for D1; and 333.3, 125.0 and 111.1 DD for D2 respectively.

**Fig 9:**
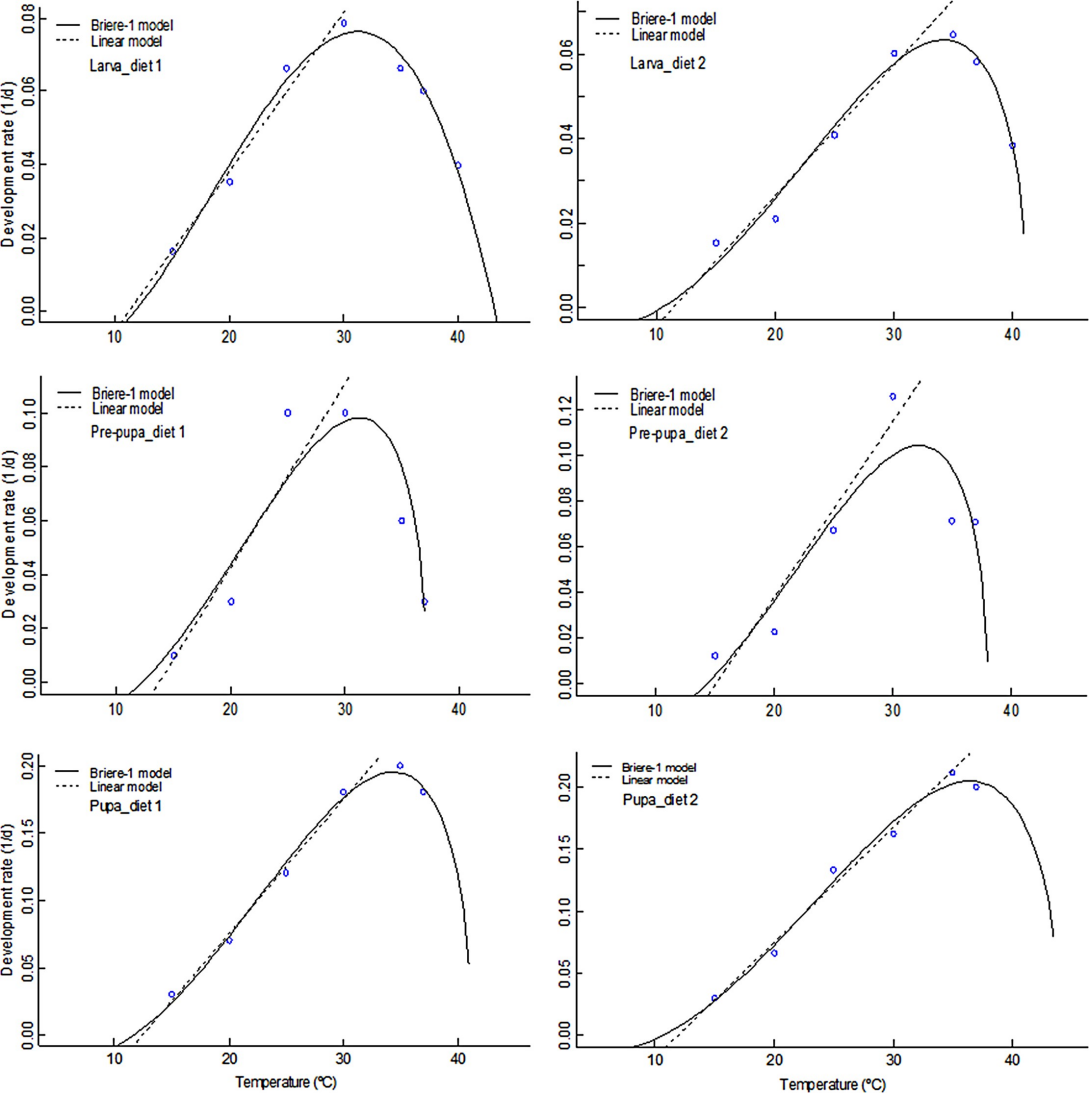
Linear and non-linear models fitted to observed values of the development rate of black soldier fly life stages at constant temperatures.

**Table 2:** Estimates of linear model parameters describing the relationship between temperature and developmental rate of the black soldier fly eggs.

Model Parameter	Estimate (± SE)
*a*	-0.269 ± 0.067
*b*	0.011 ± 0.002
*T*_*min*_	13.647 ± 1.98
*k*	50.736 ± 5.751
*RSS*	0.013
*R*^*2*^	0.940

**Table 3:** Estimates of model parameters describing the relationship between temperature and developmental rate of black soldier fly life stages on two diets.

Model	Model Parameters	Larva	Pre-pupa	Pupa
**D1**				
Linear	*a*	-0.049 ± 0.011	-0.093 ± 0.042	-0.093 ± 0.015
	*b*	0.004 ± 0.001	0.007 ± 0.002	0.007 ± 0.001
	*T*_*min*_	12.25 ± 1.41	13.29 ± 2.65	13.29 ± 1.92
	*k*	250.2 ± 25.50	142.86 ± 39.17	142.86 ± 39.17
	*RSS*	5.83 x 10^−5^	8.20 x 10^−4^	8.20 x 10^−4^
	*R*^*2*^	0.976	0.876	0.876
Brière-1	*n*	2.09 x10^-5^	6.77 x10^-5^	9.59 x10^-5^
	*T*_*min*_	11.74 ± 0.94	12.17 ± 3.90	11.75 ± 1.08
	*T*_*max*_	43.23 ± 0.47	37.18 ± 0.29	41.21 ± 0.81
	*T*_*opt*_	35.99	31.26	34.39
**D2**				
Linear	*a*	-0.035 ± 0.011	-0.117 ± 0.036	-0.110 ± 0.024
	*b*	0.003 ± 0.001	0.008 ± 0.002	0.009 ± 0.001
	*T*_*min*_	11.67 ± 3.00	14. 63 ± 1.83	12.22 ± 1.67
	*k*	333.33 ± 50.59	125.00 ± 25.88	111.11 ± 11.90
	*RSS*	5.80 x 10^−5^	5.91 x 10^−4^	2.58 x 10^−4^
	*R*^*2*^	0.954	0.926	0.976
Brière-1	*n*	2.93 x 10^−5^	7.50 x 10^−5^	7.92 x10^-5^
	*T*_*min*_	10.39 ± 1.68	14.28 ± 3.41	10.66 ± 1.96
	*T*_*max*_	41.22 ± 0.34	38.02 ± 0.97	43.99 ± 2.34
	*T*_*opt*_	34.20	32.25	36.44

D1 = diet 1, D2 = diet 2

### Estimated life table parameters of *H*. *illucens* reared on two diets at different temperatures

The net reproductive rate (*R*_*o*_) (F = 4.57; df = 3,13; *P* = 0.021 for D1 and F = 5.79; df = 3,13; *P* = 0.010 for D2), intrinsic rate of increase (*r*_*m*_) (F = 26.91; df = 3,13; *P* < 0.0001 for D1 and F = 30.68; df = 3,13; *P* < 0.0001 for D2), gross reproductive rate (GRR) (F = 3.33; df = 3,13; *P* = 0.053 for D1 and F = 3.86; df = 3,13; *P* = 0.036 for D2) and finite rate of increase (λ) (F = 25.36; df = 3,13; *P* < 0.0001 for D1 and F = 28.95; df = 3,13; *P* < 0.0001 for D2) values were significantly higher at 30°C, when compared to the other temperature treatments (Table 4). The doubling time (*T*_*d*_) (F = 28.14; df = 3, 13; *P* < 0.0001 for D1 and F = 29.64; df = 3, 13; *P* < 0.0001 for D2) and mean generation time (G) (F = 751.60; df = 3, 13; *P* = 0.0001 for D1 and F = 11.84; df = 3, 13; *P* = 0.0001 for D2) at 30°C was significantly shorter compared to the other temperature treatments. The values of *R*_*o*_ generated for all the temperature regimes and diets indicated significant growth of *H*. *illucens* population rather than a decline. In addition, for all temperature treatments and diets, analysis of life table and fecundity parameters yielded positive values of intrinsic rate of increase, implying population growth.

**Table 4:** Life table parameters of black soldier fly reared on two different diets at constant temperatures.

Parameter	20°C		25°C		30°C		35°C	
	D1	D2	D1	D2	D1	D2	D1	D2
Intrinsic rate of increase (*r*_*m*_) (daughters per female per day)	0.043 ± 0.002cA	0.036 ± 0.002cA	0.098 ± 0.005bA	0.070 ± 0.004bB	0.127 ± 0.014aA	0.122 ± 0.013aA	0.109 ± 0.005bA	0.108 ± 0.005bA
Doubling time (*T*_*d*_) (days)	16.25 ± 2.50aA	19.55 ± 3.12aA	7.10 ± 0.63bA	9.86 ± 0.91bB	5.46 ± 0.99bA	5.67 ± 1.00bA	6.39 ± 0.73bA	6.43 ± 0.78bA
Net reproductive ratio (*R*_*0*_)	116.10 ± 2.96bA	105.80 ± 2.70bA	97.66 ± 5.06bA	66.27 ± 3.43bA	186.93 ± 22.52aA	196.26 ± 23.64aA	118.07 ± 4.26bA	102.33 ± 3.69bA
Gross reproductive ratio (*GRR*)	190.32 ± 4.86bA	179.32 ± 4.57bA	145.77 ± 7.55abA	144.07 ± 7.46bA	242.76 ± 29.24aA	261.68 ± 31.52aA	178.90 ± 6.45bA	189.50 ± 6.83bA
Mean generation time (*G*) (days)	111.91 ± 1.24aA	131.46 ± 1.24aB	46.65 ± 1.16bA	59.65 ± 1.16bB	41.18 ± 2.52bA	43.18 ± 2.52cA	43.95 ± 0.71bA	42.95 ± 0.71cA
Finite rate of increase (λ)	1.044 ± 0.002cA	1.036 ± 0.002cA	1.103 ± 0.005bA	1.073 ± 0.004bB	1.135 ± 0.016aA	1.130 ± 0.015aA	1.115 ± 0.005bA	1.114 ± 0.005bA

D1 = diet 1, D2 = diet 2. Means within the same row followed by different lower-case letters are significantly different (*P* < 0.05, SNK Test) for D1 or D2. Means within the same row for each temperature regime followed by the same upper-case letters are not significantly different (*P* < 0.05, T-test) for D1 and D2.

## Discussion

The rate of black soldier fly growth and development was considerably influenced by temperature and diet, which are the two most critical environmental factors. Previous studies have shown that insects including the BSF are sensitive to several environmental factors, especially temperature, which is considered the most important abiotic factor [48] that can impact not only insect developmental rate, seasonal and daily cycles [49] but also, indirectly effects different aspects of the insect biology, such as immature survival, adult life span, growth, fecundity, fertility, sex ratio and, population growth parameters [50–52]. As such, temperature would profoundly influence the behaviour, abundance, colonization, distribution, life table parameters and fitness of the insect species. Thus, knowledge generated on thermal requirements of *H*. *illucens*’ development will have significant implications for production programs, as the population growth and population size as well as their differences under divergent conditions is determined. Thus, life history studies would provide successful ways of following up changes in a population’s growth and several other important aspects of the insect’s life cycle that are temperature-dependent [53–57]. Thus, information regarding the population structure becomes crucial in achieving optimum range of temperatures, which would help in boosting the insect population for optimal mass production [52].

Here, we used nine different constant rearing temperatures to evaluate the influence that different temperature regimes would have on black soldier fly development. Lower (<15°C) and upper (>40°C) temperature development thresholds were fatal to the insects given that all the eggs completely failed to hatch at these temperatures. Our results confirmed the sensitivity of BSF to extremely low and high temperatures as indicated in previous studies [17, 19]. Egg viability and hatchability observed in this study occurred between 15–40°C. Our observation at lower temperature threshold concurs with that presented by [17] at 16°C. The levels of egg viability at 15 and 40°C were extremely low (< 12%), which is consistent with the report by [17] at 16°C. Successful egg eclosion reported by [17] at 19°C was equivalent to high egg eclosion recorded in this present study at 30 and 35°C with survival of 80 and 75%, respectively. This present result is consistent with the results obtained by Holmes et al. [58]. Contrary to observations by [17], who reported that newly hatched larvae of BSF failed to survive at 16°C, we found that BSF young larvae at 15°C did not die after hatching, suggesting that lower developmental threshold for BSF eggs is at 15°C. Thus, hatchability or egg eclosion could be a pointer to colony efficiency under standard conditions in any mass-rearing systems [59].

According to previous studies, nutrient quality of larval food has been shown to have considerable influence on the amount and quality of emerged adult flies, which indirectly affects the developmental duration, survival and growth of the black soldier larvae [19, 60–63]. The two types of diet tested in this study successfully supported the development of *H*. *illucens*, although the production of adult flies varied significantly with the diet type used. All BSF life stages (larval, pre-pupal and pupal) completed development to adults in the range of 15–37°C. Survivorship rates of BSF immature life stages differed remarkably at different temperature regimes. Temperature treatments below 15°C and above 40°C were unfavourable with complete mortality of *H*. *illucens* life stages observed. The present studies showed that the upper temperature threshold of the larval stage was 40°C with survival of 34 and 28% on D1 and D2, respectively. Only 5 and 20% of the flies from D1 and D2, respectively, at 37°C emerged as adults, many of the flies showed signs crippling malformations (i.e. could neither walk nor fly normally and were unable to feed). At 40°C, none of the pre-pupal stages successfully completed development to proceed to pupal stage on the various diets. These findings slightly deviate from those reported by [19], who showed that although 73% of *H*. *illucens* larvae were able to develop and survive to pre-pupae (post-feeding stage) at 36°C, only 0.1% of pupae successfully developed and emerged as adults, thus implying an upper developmental temperature threshold at 36°C. The reasons for these differences between the two studies are unknown but nutrient content of diet types, geographical strains of the two populations (Kenyan and Texas, USA populations) and adaptations of both populations of *H*. *illucens* might have contributed to the observed variation. Furthermore, this is supported by the studies carried out by [64, 65], which revealed that insect populations from various geographical landscape may vary in their reproductive fitness and life table traits [66].

The linear relationship between the developmental rates and temperature for rearing BSF life was positive. The estimated lower temperature developmental threshold (LTDT) of BSF eggs according to the linear model was 13.65°C. Here, we publish the first report on lower developmental threshold temperatures of BSF immature life stages, which are consistent with reports of other dipteran species like tephritid fruit flies [23, 28]. The divergence observed between the different reported LTDTs may be attributed to variations in rearing conditions and possible feedstocks utilized as food for the BSF larvae [67]. Unlike other studies conducted, furthermore to the LTDTs, we also established the optimal temperatures for development as well as upper development threshold temperatures for the different life stages by fitting the non-linear functions of higher biological significance. The optimum developmental temperature range for *H*. *illucens* life stages reared on D1 was 31.3–36. 0°C and 32.3–36.4°C on D2. In literature, there are no studies reported with regards to upper developmental threshold or lethal upper temperature thresholds of BSF, which according to the present studies were estimated to range between 37.2–44.0°C for the various developmental stages. Fatal temperature thresholds established in the current studies may be applicable for future mass production technology programs for BSF. The present findings revealed that the eggs required 69 degree-days (DD) to complete development to the next stage (larval stage). Total degree-days required for BSF larval stages to successfully change to the pre-pupal stage was recorded at developmental temperature threshold of 10.4–14.3°C, which was higher (250–333 DD) compared to the other stages (< 150 DD). Several authors have also reported huge discrepancies in thermal requirements for different insect species and immature life stages, which can be ascribed to differences in methodological approaches or aspects related to larval food quality and quantity as well as larval density in the rearing facilities [28, 29, 68, 69]. It is worth noting that insects require fewer degree days for development when fed on high quality food resources compared to low quality resources [70], which explains the considerable variation observed when the larvae were provided the two types of diets.

Pronounced quality control parameters observed in this study included high pupal recovery and heavier puparia when the fly larvae fed on D1, which was supplemented with brewers’ yeast compared with those fed on D2 (not supplemented). It is important to note that the pupal mass of insects have been demonstrated to be a practical quality control benchmark in insect mass rearing facilities, where size has been observed to show strong correlation with male flies mating successes [71]. Furthermore, the insect pupal weight has been widely used as an estimate of size, [71] and revealed that adult flies eclosing from heavy puparia showed higher mating success than those with lower weight. Also, large flies have been shown to demonstrate better flight capability than smaller flies [72]. Heavy female insects have also been shown to have a proportionately higher lifetime fecundity compared to light females [73]. This implies that increased pupal weights will produce larger individuals (adults). Thus, from a commercial point of view, this highlights the significance of safeguarding maximum pupation while optimising female-to-male ratio to promote healthy mating behaviour within the insect stock colonies [59, 74, 75].

Black soldier fly fecundity recorded was an important parameter, which was observed to be directly related to differences in the temperature regimes. This attribute has been documented in different insects, where an increasing swift in rearing temperatures frequently result in visible decreases in female flies productivity or complete cessation of egg production [76, 77], as realized fecundity could have been restricted by both temperature dependency of egg maturation and oviposition [78]. The present study demonstrates that adult female BSF reared on the two diets were capable of reproducing between the temperature range of 20–35˚C. We also observed increased physical inactivity of larvae and reduced feed intake followed by death with increase in temperature (>35˚C). The male and female flies’ lifespan gradually decreased with increased temperature from 15 to 37˚C. Thus, at temperatures above 35°C, the longevity of adult female flies was 4–5 times less compared to those at 15°C with shortened reproductive phase. The life time fecundity of black soldier flies was temperature-dependent revealing a curvilinear response curve for fecundity to reach its maximum at 30°C with subsequent decrease at temperature levels below and above this temperature. This implies that the prevailing optimal temperature recorded in the present study can play a major role in defining climatic fitness for mating and oviposition of adult black soldier flies. The phenomenon of decreased female fecundity have become noticeable in different insects in the face of temperature increases [76, 77]. A comparable trends in temperature-dependent fecundity have been observed for different insects with temperatures between 25–30°C reported as a suitable choice for reproduction, whereas temperatures above 35°C remained extremely unfavourable [25]. Our study reports for the first time the effect of temperature on *H*. *illucens’* fecundity with slightly higher number of eggs oviposited per female throughout its lifespan in comparison to that documented in literature [79]. This could be attributed to the dissimilar ovipositional behaviour response of BSF when fed on various diets [79]. Oviposition pattern of insect is an important life table component, thus comprehensive information on temperature-driven age-specific off-spring production is critical for generating models that forecast BSF population growth [80]. However, fecundity is affected by many factors, which include larval and adult density, availability of feedstock, nutrition of immature life stages, and several environmental factors, which need to be studied further to improve BSF mass-production.

Life-table data offer readily available means of tracking population growth as well as other changes [81], and remain the most powerful tools for analyzing and understanding the impact that abiotic factors have upon the growth, survival, reproduction and rate of increase of an insect population [82, 83]. The impact of temperature on BSF life table parameters is reported here for the first time and describes a series of temperature regimes, which are within the ecologically niche suitability limits related to BSF development, establishment and colonization. These findings provide answers to the optimum developmental threshold temperature conditions of BSF mass production with 30°C being the most favorable temperature with considerable higher intrinsic rate of natural increase and shorter doubling time. Thus, the implication of using fertility tables to improve mass rearing conditions has been reported for many insect species by assessing the variations in their reproductive rate and the total number of female offspring produced per female per generation [84]. The intrinsic rate of natural increase is extremely essential as its describes the population increase of the insects in an unrestricted environment, basically addressing the differences between birth rate and death rate [84] when the insects are exposed to various food sources [85–90].
